# A Decoupled Variational Bayesian Adaptive Kalman Filter for MEMS INS/GNSS Integrated Navigation Under Time-Varying Noise Covariances

**DOI:** 10.3390/s26216888

**Published:** 2026-10-30

**Authors:** Kaiqiang Feng, Jie Li, Zhirui Sun

**Affiliations:** State Key Laboratory of Extreme Environment Optoelectronic Dynamic Measurement Technology and Instrument, North University of China, Taiyuan 030051, China; lijie@nuc.edu.cn (J.L.); bz202506005@st.nuc.edu.cn (Z.S.)

**Keywords:** MEMS INS/GNSS integrated navigation, adaptive Kalman filter, covariance matching, time-varying noise covariance, Bayesian model averaging, normalized innovation squared

## Abstract

We evaluate a decoupled variational Bayesian adaptive Kalman filter (D-VBAKF) for MEMS INS/GNSS navigation, combining bounded fading-memory covariance matching for R with motion-regularized Bayesian model averaging over scaled Q candidates. In 50 paired trials of a 15-state, 120 s prescribed-noise simulation, 3D position RMSE reductions versus EKF-Fixed were 2.94 m (nominal paired 95% interval [1.59, 4.29] m) for 10 s outages and 7.76 m ([1.51, 14.02] m) for 30 s outages. At 60 s, intervals versus EKF-Fixed and joint-VB VBAKF included zero; all 90 s intervals included zero. In the separate no-outage simulation, Q-KL was 0.011, and the R relative error was 0.173. On one 1617 s ICM20602 drive with deterministic GNSS perturbations, full-route 3D RMSE was 27.19 m versus 30.77 m for EKF-Fixed. Across twelve repeated outage windows on that drive, the R-only variant yielded 28.19 m versus 28.86 m for the full method; BMA-Q only yielded 33.98 m versus 33.23 m for EKF-Fixed. Thus, the real-data experiment did not demonstrate an incremental accuracy benefit from adding BMA-Q to R-only adaptation, and validation on a single drive limits the generalizability of the findings. NIS coverage was 0.636 for D-VBAKF and 0.626 for EKF-Fixed, both below the nominal 0.95.

## 1. Introduction

Micro-electro-mechanical-system strapdown inertial navigation system (MEMS-SINS) and global navigation satellite system (GNSS) integration is widely used for vehicle and mobile-platform navigation [1,2,3,4]. The MEMS-SINS provides high-rate attitude, velocity, and position increments without reliance on external signals, whereas the GNSS corrects the long-term drift caused by inertial-sensor biases. Owing to these complementary characteristics, the integrated navigation filter is central to obtaining stable and accurate navigation information.

Kalman filtering and its nonlinear extensions are among the most common estimation strategies for INS/GNSS data fusion [3,5,6]. However, Kalman-filter performance depends strongly on accurate prior knowledge of the process-noise and measurement-noise covariances [6,7,8]. This assumption is difficult to satisfy in low-cost MEMS INS/GNSS systems. The effective process-noise model can require adjustment when vibration, temperature-dependent inertial errors, or unmodeled vehicle dynamics make the fixed error-state model inaccurate [1,9,10,11]. GNSS measurement errors may change with satellite geometry, multipath, and non-line-of-sight reception; complete blockage produces an unavailable measurement [2,4,12]. Under such conditions, fixed-covariance matrices can make the filter covariance inconsistent and may degrade navigation accuracy or even cause divergence.

Adaptive Kalman filtering methods have been extensively investigated to address stochastic-model uncertainty. Innovation-based adaptive filters and covariance-matching methods estimate noise statistics by forcing the innovation sequence to match its theoretical covariance [6,13,14,15,16]. These methods are straightforward to implement and computationally efficient, but covariance estimation often requires a sliding window. A large window improves statistical reliability but limits responsiveness to rapidly changing noise. Multiple-model adaptive estimation represents process uncertainty through a model bank [17,18,19,20,21], but its performance depends on whether the model set adequately covers the true stochastic behavior. Strong-tracking and fading-factor filters enlarge the predicted covariance to improve tracking capability; however, the fading factor is often chosen empirically and may not yield a reliable estimate of the actual covariance [22].

Variational Bayesian (VB) adaptive Kalman filtering provides another framework for online estimation of unknown covariance matrices [23,24,25,26]. Conjugate covariance priors, including inverse-Wishart models, permit recursive probabilistic updates [26,27]. Here, $Q_{k}$ denotes the process-noise covariance in the inertial error-state prediction, and $R_{k}$ denotes the measurement-noise covariance of the GNSS update. Estimating both as unrestricted, time-varying matrices from an instantaneous innovation covariance is poorly conditioned: the observed covariance combines $H_{k}P_{k|k-1}H_{k}^{T}$ and $R_{k}$, while $P_{k|k-1}$ depends on $Q_{k-1}$ through the covariance-prediction (Riccati) recursion [7]. A change in the process contribution can therefore be offset by a change in $R_{k}$, leaving the same innovation covariance [13,28,29]. Temporal information or structural constraints can help distinguish the two; Section 3.3 makes the single-epoch ambiguity explicit. This issue matters in MEMS INS/GNSS navigation because inertial-model uncertainty and GNSS degradation can occur during the same motion segment. This paper evaluates a decoupled VB-motivated adaptive Kalman filter (D-VBAKF) for MEMS INS/GNSS integrated navigation under time-varying noise covariances. Its R channel uses bounded fading-memory covariance matching conditional on the selected predicted covariance; its Q channel uses motion-regularized Bayesian model averaging (BMA) over scaled candidates. An inverse-Wishart VB update is presented as a reference model in Section 3.4. The contributions are as follows:

1. A conditional innovation-covariance identity and compensating-perturbation relation characterize unrestricted single-epoch Q/R allocation.

2. The implemented filter combines conditional covariance matching for *R* with motion-regularized BMA over six scalar multiples of $Q_{0}$. The resulting process covariance changes scale but not shape.

3. The evaluation uses 50 paired prescribed-noise simulation trials and one public vehicle drive with deterministic GNSS stressors. It reports paired simulation RMSE intervals, no-outage covariance tracking, NIS coverage, update time, and component ablation across twelve windows on the drive.

## 2. Related Work and Research Gap

Adaptive filtering for INS/GNSS integrated navigation uses several information sources. Innovation-based covariance matching uses recent residual statistics and is attractive for online navigation because of its recursive structure [6,14,15,16]. A short averaging window has high sampling variability; a longer window smooths estimates but responds more slowly to changing noise. Multi-lag innovation autocovariances and structured models can provide additional information for covariance identification when their excitation and rank conditions hold [13,28,29,30,31].

Covariance inflation and fading-factor filters enlarge the predicted covariance to improve maneuver tracking [8,22]. The fading factor primarily acts on predicted-state covariance and does not by itself identify the process-noise or measurement-noise covariance. Recent work also addresses process-covariance estimation in inertial navigation through adaptive selection and sample screening [8,32,33]. Stochastic MEMS modeling and GNSS-denied filtering provide related application settings [11,34].

Probabilistic covariance learning offers a structured uncertainty framework. EM and variational Bayesian adaptive Kalman filtering update unknown model parameters using likelihoods and priors [23,24,25,26,35]. Multiple-model and Bayesian model averaging methods instead select among candidate stochastic models [17,18,20,26]. Their performance depends on the prior model family; the scaled Q bank considered here can change overall magnitude but not covariance shape. Bilgin and Olivier combined inverse-Wishart measurement-noise learning with process-model averaging in nonlinear Kalman filtering [26]. The present implementation uses conditional fading-memory covariance matching for R and a fixed six-scale Q bank in MEMS INS/GNSS navigation.

Unrestricted Q and R can produce the same instantaneous innovation covariance [13,26,28,29]. Multi-lag information or structural constraints can distinguish some allocations [28,30,31]. The evaluated implementation uses conditional R estimation and a scaled Q bank for MEMS INS/GNSS navigation.

## 3. Materials and Methods

### 3.1. Problem Formulation and Workflow

**Figure 1:**
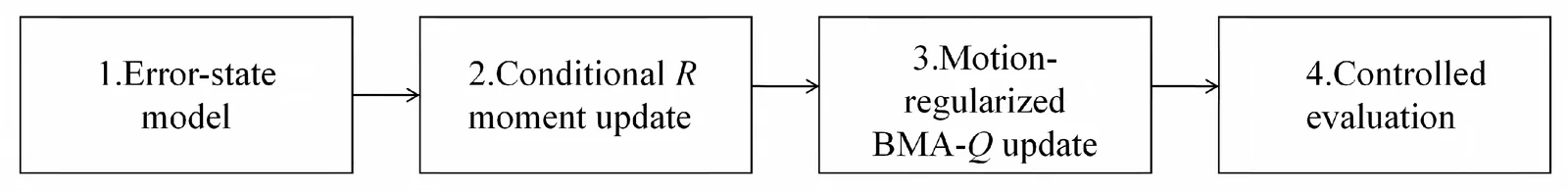
Four stages of this study.

This study follows four stages, as shown in Figure 1.

As shown in Figure 1: First, a loosely coupled MEMS INS/GNSS error-state model is established. This fixes the deterministic filtering structure so that stochastic-model adaptation can be analyzed separately.

Second, the time-varying covariance problem is analyzed. The innovation covariance gives a conditional decomposition of R after the predicted covariance has been selected; one finite-sample innovation does not identify its population value.

Third, D-VBAKF applies a bounded conditional covariance-matching R update and motion-regularized Bayesian model averaging over a scalar Q bank. Fourth, the reported comparisons use the same model bank, covariance bounds, forgetting factors, and weight safeguards within each evaluation protocol.

The proposed method is compared with representative filters under paired simulated trajectories and noise realizations. A separate public-data test uses one recorded vehicle trajectory with imposed GNSS stressors. Navigation error, known-covariance tracking in simulation, measurement-space NIS, and update time are reported; NIS alone does not establish state-covariance consistency.

### 3.2. MEMS INS/GNSS Integration Model

#### 3.2.1. Error-State Definition

For a loosely coupled MEMS INS/GNSS integrated navigation system, the error-state vector is defined as

(1) $$x_{k}={\left[\begin{array}{c} \delta p_{k}^{T},\delta v_{k}^{T},\psi_{k}^{T},b_{g,k}^{T},b_{a,k}^{T} \end{array}\right]}^{T},$$

where $\delta p_{k}$, $\delta v_{k}$, $\psi_{k}$, $b_{g,k}$ and $b_{a,k}$ denote the position error, velocity error, small attitude error, gyro bias error and accelerometer bias error, respectively. The dimension of the state vector is 15. This state definition separates fast navigation-error dynamics from slowly varying inertial-sensor biases.

#### 3.2.2. State Propagation Model

The discrete-time linearized error-state propagation model is expressed as

(2) $$x_{k}=F_{k-1}x_{k-1}+w_{k-1},$$

where $F_{k-1}$ is the state-transition matrix, and $w_{k-1}∼N\left(0,Q_{k-1}\right)$ is the process noise. The corresponding Kalman prediction equations are

(3) $${\hat{x}}_{k|k-1}=F_{k-1}{\hat{x}}_{k-1|k-1},$$

(4) $$P_{k|k-1}=F_{k-1}P_{k-1|k-1}F_{k-1}^{T}+Q_{k-1}.$$

Here $P_{k|k-1}$ is the predicted error-state covariance at epoch *k*, and $P_{k-1|k-1}$ is the posterior error-state covariance after the preceding update [7]. In a low-cost MEMS system, $Q_{K-1}$ represents residual inertial-sensor uncertainty, unmodeled bias variation, vibration-induced model mismatch, and discretization error. If this covariance is underestimated, the filter becomes overconfident in inertial propagation. If it is overestimated, excessive uncertainty may be injected into the navigation solution.

#### 3.2.3. GNSS Measurement Model

The GNSS measurement equation is written as

(5) $$z_{k}=H_{k}x_{k}+v_{k},$$

where $H_{k}$ maps the error state to the GNSS position and velocity measurement residual, and $v_{k}∼N\left(0,R_{k}\right)$ is the measurement noise. In the benchmark used in this paper, $z_{k}$ is a six-dimensional vector containing three position residuals and three velocity residuals. The measurement covariance $R_{k}$ characterizes GNSS observation quality and may vary with satellite geometry, multipath, masking, receiver tracking status, and interference.

#### 3.2.4. Innovation Statistics

The innovation of the Kalman filter is

(6) $$\nu_{k}=z_{k}-H_{k}{\hat{x}}_{k|k-1},$$

and its theoretical covariance is

(7) $$S_{k}=H_{k}P_{k|k-1}H_{k}^{T}+R_{k}.$$

The innovation sequence is the principal online statistic available for covariance adaptation. However, $S_{k}$ contains both the predicted-state covariance and the measurement covariance. Because $P_{k|k-1}$ depends on Q through the Riccati covariance recursion [7], one instantaneous innovation covariance alone cannot uniquely separate process-side uncertainty from measurement-side uncertainty [13,28,29].

### 3.3. Time-Varying Noise Covariance Problem

#### 3.3.1. Sources of Time-Varying *Q* and *R*

The process-noise covariance Q is a model for uncertainty in the inertial error-state propagation; it need not equal a physical white-noise level measured in the laboratory. MEMS sensor errors can vary with temperature and vibration [9,10]. Rapid maneuvers can also increase the effective model error if the fixed linearization or bias model does not represent the motion adequately [1]. In these conditions, a fixed nominal Q may understate or overstate prediction uncertainty.

For available GNSS observations, multipath, degraded satellite geometry, and non-line-of-sight reception can alter measurement uncertainty and may introduce bias or correlation [2,4,12]. A complete signal blockage yields no GNSS measurement and is treated as an outage here. A fixed R can cause the filter to overtrust degraded observations or underuse reliable ones. Simultaneous adjustment of Q and R from the same instantaneous innovation information is more difficult because both contribute to its covariance.

#### 3.3.2. Conditional Covariance Decomposition for R

If $P_{k|k-1}$ is fixed at epoch *k*, the population innovation covariance algebraically defines a measurement-side contribution:

(8) $$R_{k}=S_{k}-H_{k}P_{k|k-1}H_{k}^{T}.$$

This is a conditional covariance decomposition, not a proof that R is statistically identifiable from one finite-sample innovation. Sampling error in an estimate ${\hat{S}}_{k}$ and error ΔP in the selected predicted covariance enter the raw estimate as ${\hat{R}}_{k}-R_{k}=\left({\hat{S}}_{k}-S_{k}\right)-H_{k}\Delta PH_{k}^{T}$. Bounds and forgetting may limit transient numerical effects but cannot remove this dependence.

#### 3.3.3. Instantaneous Ambiguity in Unrestricted Q/R Adaptation

At one update, unrestricted Q and R cannot generally be distinguished from the instantaneous innovation covariance alone. With the previous posterior covariance held fixed, a perturbation ΔQ changes the predicted covariance by ΔQ and produces

$$\Delta S_{k}=H_{k}\Delta QH_{k}^{T}+\Delta R_{k}.$$

Choosing $\Delta R_{k}=-H_{k}\Delta QH_{k}^{T}$ leaves $S_{k}$ unchanged whenever the perturbed covariance matrices remain admissible. Thus, the same instantaneous statistic can support different Q/R allocations. Innovation autocorrelations across time, independent sensor information, or structural restrictions may supply the additional information needed for identification [13,28,29]; the ambiguity described here concerns an unrestricted same-epoch update.

Without that additional information, an unrestricted same-epoch joint update can favor an extreme allocation in which Q is too small, and R is too large, or vice versa. Such an allocation may yield acceptable state estimates in some trials while distorting posterior uncertainty. The finite Q model bank and the conditional R update restrict the range of possible allocations.

**Remark** **1.** *The method addresses this single-epoch allocation ambiguity by conditioning R on the selected predicted covariance and constraining Q to a scaled model bank with a motion-informed regularization prior. Identification from richer temporal data is a separate question.*

### 3.4. Proposed D-VBAKF Framework

#### 3.4.1. Decoupled Adaptation Principle

D-VBAKF adapts the two covariances asymmetrically. The measurement-noise covariance R is estimated conditionally after a predicted covariance has been selected. The process-noise covariance Q is represented by scaled candidates and adapted through Bayesian model averaging. This is an algorithmic regularizer of the same-epoch allocation problem, not a proof of statistical identifiability or physical independence between Q and R.

At each GNSS measurement epoch, D-VBAKF performs four sequential operations: (i) use the current BMA process covariance for the time update; (ii) use the innovation moment to update R conditionally; (iii) evaluate the predictive likelihood of each Q candidate using the updated R; and (iv) compute the BMA process covariance for the next prediction. This structure preserves feedback between Q and R while avoiding unrestricted joint covariance fitting.

Let $m_{k}\in \left\{1,\ldots ,M\right\}$ denote the process-noise model index, where $Q_{k}=Q_{k}^{\left(m_{k}\right)}$. The implemented filter propagates a state estimate and covariance, a bounded point estimate ${\hat{R}}_{k}$, and discrete model weights $w_{k}^{\left(m\right)}$. It does not maintain a variational distribution $q(R_{k})$ or optimize an evidence lower bound. The inverse-Wishart equations below state the VB reference model; the operational R rule is the covariance-matching estimator that follows them. Errors in the selected $P_{k|k-1}$ enter this estimator through the subtraction of $H_{k}P_{k|k-1}H_{k}^{T}$.

For the process covariance, D-VBAKF updates the discrete model probability by predictive evidence and a motion regularizer:

(9) $$p\left(m_{k}|Z_{k}\right)\propto p\left(z_{k}|m_{k},Z_{k-1},{\hat{R}}_{k}\right)p\left(m_{k}|Z_{k-1}\right)p_{\mathrm{phys}}\left(m_{k}|t_{k}\right).$$

Thus, R is estimated conditionally as a bounded covariance, whereas Q is adapted through probabilities over a finite covariance set. This discrete model-weight update is not variational inference.

**Proposition** **1.** *If every candidate $Q_{k}^{\left(m\right)}$ is symmetric positive definite, $w_{k}^{\left(m\right)}>0$, and $\sum_{m=1}^{M}w_{k}^{\left(m\right)}=1$, then the BMA covariance ${\bar{Q}}_{k}=\sum_{m=1}^{M}w_{k}^{\left(m\right)}Q_{k}^{\left(m\right)}$ is symmetric positive definite. The proof follows directly from convexity. For any nonzero vector a*,

(10) $$a^{T}{\bar{Q}}_{k}a=\sum_{m=1}^{M}w_{k}^{\left(m\right)}a^{T}Q_{k}^{\left(m\right)}a>0.$$

The entropy floor introduced in Section 3.5 further ensures that no candidate weight becomes permanently zero. This positive-definiteness property is important because it guarantees that the time-update covariance remains a valid covariance matrix after process-noise adaptation.

#### 3.4.2. Conditional Measurement-Covariance Update

As a VB reference model, consider assigning the measurement-noise covariance an inverse-Wishart prior,

(11) $$R_{k}∼\mathrm{IW}\left(\nu_{k|k-1},V_{k|k-1}\right),$$

where $\nu_{k|k-1}$ and $V_{k|k-1}$ are the degrees of freedom and scale matrix, respectively. A diagonal covariance form is adopted because the simulated GNSS position and velocity channels are generated as uncorrelated components. The diagonal form also improves numerical stability when only one innovation sample is available at an epoch. For this reference model, the mean-field update is

(12) $$\log q^{*}\left(R_{k}\right)=E_{q(x_{k})}\left[\log p\left(z_{k}|x_{k},R_{k}\right)\right]+\log p\left(R_{k}\right)+C,$$

where *C* is a normalization constant independent of $R_{k}$. Since the measurement likelihood is Gaussian and the prior is inverse-Wishart, the optimal variational distribution remains inverse-Wishart:

(13) $$q^{*}\left(R_{k}\right)=\mathrm{IW}\left(\nu_{k|k-1}+1,V_{k|k-1}+\Psi_{k}\right),$$

with

(14) $$\Psi_{k}=E_{q(x_{k})}\left[\left(z_{k}-H_{k}x_{k}\right){\left(z_{k}-H_{k}x_{k}\right)}^{T}\right].$$

This conjugate update shows how an inverse-Wishart VB reference model incorporates the expected posterior residual second moment [26,27]. These inverse-Wishart parameters and the distribution $q^{*}\left(R_{k}\right)$ are not propagated in the reported algorithm. The implemented diagonal estimator below instead discounts the innovation second moment, subtracts the selected predicted-state contribution, and applies covariance bounds. It is a conditional covariance-matching rule, not the mean of the inverse-Wishart posterior above.

For the implemented diagonal approximation, let the discounted innovation moment be

(15) $${\hat{C}}_{\nu ,k}=\lambda_{R}{\hat{C}}_{\nu ,k-1}+\left(1-\lambda_{R}\right)\mathrm{diag}\left(\nu_{k}\nu_{k}^{T}\right).$$

The corresponding conditional measurement-side estimate is

(16) $${\hat{R}}_{k}^{\mathrm{CM}}=\mathrm{diag}\;\left({\hat{C}}_{\nu ,k}-H_{k}P_{k|k-1}H_{k}^{T}\right),$$

and the bounded update used by the filter is

(17) $${\hat{R}}_{k}={\mathrm{clip}}_{R}\;\left({\hat{R}}_{k}^{\mathrm{CM}}\right).$$

The superscript CM denotes covariance matching and distinguishes the implemented moment approximation from the VB reference model. Here diag retains the diagonal entries and zeros the off-diagonal entries, and ${\mathrm{clip}}_{R}$ applies the stated bounds to those entries. Conditioning on the already selected $P_{k|k-1}$ limits simultaneous assignment of innovation energy to both Q and R. The update is recursive and does not store an innovation window. The lower bound prevents the estimated GNSS measurement variance from falling below its nominal value; it does not establish calibration of the predicted or posterior state covariance. The upper bound caps the measurement-variance estimate after an abnormal innovation.

The resulting ${\hat{R}}_{k}$ is conditional on the process model that produced $P_{k|k-1}$; it is not an unconditional estimate of physical GNSS noise. A different selected process covariance changes the subtraction term for the same observed innovation moment. Forgetting and clipping do not eliminate this coupling.

#### 3.4.3. Prior-Regularized BMA Adaptation of Process Covariance R

Following model averaging and multiple-model filtering [17,18,20,26], the process covariance is represented by *M* scaled candidates:

(18) $$Q_{k}^{\left(m\right)}=\alpha_{m}Q_{0},\;m=1,\ldots ,M,$$

where $Q_{0}$ is the nominal process covariance and $\alpha_{m}$ is the scale factor. For a candidate $Q_{k}^{\left(m\right)}$, the predicted covariance and candidate innovation covariance are

(19) $$P_{k|k-1}^{\left(m\right)}=F_{k-1}P_{k-1|k-1}F_{k-1}^{T}+Q_{k}^{\left(m\right)},$$

(20) $$S_{k}^{\left(m\right)}=H_{k}P_{k|k-1}^{\left(m\right)}H_{k}^{T}+{\hat{R}}_{k}.$$

The Gaussian predictive likelihood is

(21) $$L_{k}^{\left(m\right)}=N\left(\nu_{k};0,S_{k}^{\left(m\right)}\right).$$

Equivalently, the log-likelihood is

(22) $$\ell_{k}^{\left(m\right)}=-0.5\left[\nu_{k}^{T}{\left(S_{k}^{\left(m\right)}\right)}^{-1}\nu_{k}+\ln\left|S_{k}^{\left(m\right)}\right|+d\ln\left(2\pi \right)\right].$$

This expression shows the two terms that determine model evidence. The Mahalanobis term penalizes a candidate whose predicted innovation covariance cannot explain the observed innovation. The log-determinant term penalizes a candidate that explains the innovation only by making the covariance excessively large. Therefore, BMA is not merely a residual-minimization rule but a predictive-evidence comparison.

A motion-informed regularization prior limits the influence of a noisy single-epoch likelihood on the BMA weights:

(23) $$\rho_{k}^{\left(m\right)}=\exp\left(-\frac{{\left(\ln\alpha_{m}-\ln\alpha_{\mathrm{prior}}\left(k\right)\right)}^{2}}{2\sigma_{q}^{2}}\right).$$

In the public-data stress test, the prior center is

(24) $$\alpha_{\mathrm{prior}}\left(k\right)={\mathrm{clip}}_{\left[0.1,30\right]}\;\left[1+5\max\;\left(0,∥\frac{\Delta \theta_{k}}{\Delta t}∥-0.02\right)+0.5\left|∥\frac{\Delta v_{k}}{\Delta t}∥-g\right|\right],$$

where $\Delta \theta_{k}/\Delta t$ is the angular-rate vector in rad s^−1^, $\Delta v_{k}/\Delta t$ is the specific-force vector in m s^−2^, and *g* is standard gravitational acceleration in m s^−2^. The 0.02 rad s^−1^ threshold sets the onset of the angular-rate contribution; the coefficients 5 and 0.5 control the respective angular-rate and specific-force contributions to the dimensionless prior scale. The final clip confines that scale to the Q-bank range. These are empirical regularization settings used in the reported implementation. The retained materials contain no calibration record or parameter-wise sensitivity results that would establish their optimality or transferability. The posterior model weight is updated as

(25) $${\tilde{w}}_{k}^{\left(m\right)}={\left(w_{k-1}^{\left(m\right)}+\epsilon \right)}^{\beta }\exp\left(\ell_{k}^{\left(m\right)}\right)\rho_{k}^{\left(m\right)},$$

where β controls the memory of previous weights, ϵ prevents zero weights, and $\rho_{k}^{\left(m\right)}$ is the motion-informed regularization prior. After normalization, the process covariance used by the next filter prediction is

(26) $${\bar{Q}}_{k}=\sum_{m=1}^{M}w_{k}^{\left(m\right)}Q_{k}^{\left(m\right)}.$$

For numerical stability, the implementation uses a log-domain softmax:

(27) $$\eta_{k}^{\left(m\right)}=\beta \ln\left(w_{k-1}^{\left(m\right)}+\epsilon \right)+\ell_{k}^{\left(m\right)}+\ln\rho_{k}^{\left(m\right)},$$

(28) $$w_{k}^{\left(m\right)}=\frac{\exp(\eta_{k}^{\left(m\right)}-\eta_{\max})}{\sum_{j=1}^{M}\exp\left(\eta_{k}^{\left(j\right)}-\eta_{\max}\right)},$$

where $\eta_{\max}=\max_{j}\eta_{k}^{\left(j\right)}$. This prevents underflow when the measurement dimension is large or when the innovation is far from several candidate models.

**Proposition** **2** (Effective adaptation dimension)**.** *Because every candidate has the form $Q_{k}^{\left(m\right)}=\alpha_{m}Q_{0}$, the BMA covariance satisfies*

(29) $${\bar{Q}}_{k}=\left(\sum_{m=1}^{M}w_{k}^{\left(m\right)}\alpha_{m}\right)Q_{0}.$$

Although the normalized weight vector has M−1 free components, the resulting covariance has only one effective scale degree of freedom and lies on the scalar ray spanned by $Q_{0}$. Different weight vectors can yield the same ${\bar{Q}}_{k}$; the bank cannot adapt the covariance shape.

This restriction prevents the innovation from arbitrarily reshaping Q and makes the selected scale inspectable. It also limits the method when the true process covariance changes shape. The remaining measurement-side discrepancy enters the conditional R update.

**Remark** **2.** *The practical function of BMA-Q in this implementation is to keep process-noise adaptation within an explicit, inspectable set of six scales and to report their relative weights. Neither the selected weight nor the averaged scale identifies the exact physical covariance. The public-data ablation below does not demonstrate an incremental navigation-accuracy benefit from this component.*

#### 3.4.4. Temporal Update Logic Under GNSS Availability and Outage

D-VBAKF uses different update logic depending on GNSS availability. When GNSS measurements are available, both the conditional R update and the BMA-Q likelihood update are performed. The innovation sequence is then used to learn measurement uncertainty and reweight process-noise candidates. Let $\gamma_{k}=1$ when a valid GNSS measurement is available at epoch *k*, and $\gamma_{k}=0$ otherwise. If $\gamma_{k}=1$, D-VBAKF performs both covariance adaptation channels:

(30) $${\hat{R}}_{k}=A_{R}\left({\hat{R}}_{k-1},\nu_{k},P_{k|k-1}\right),$$

(31) $$w_{k}^{\left(m\right)}=A_{Q}\left(w_{k-1}^{\left(m\right)},\nu_{k},{\hat{R}}_{k},Q_{k}^{\left(m\right)}\right),$$

where $A_{R}\left(\cdot \right)$ denotes the bounded conditional *R* moment update, and $A_{Q}\left(\cdot \right)$ denotes the prior-regularized BMA-Q update. If $\gamma_{k}=0$, no innovation exists, so the likelihood term is undefined. In the reported implementation,

(32) $${\hat{R}}_{k}={\hat{R}}_{k-1},\;w_{k}^{\left(m\right)}=w_{k-1}^{\left(m\right)}.$$

The filter propagates only the INS error state and covariance using the current BMA process covariance. This rule avoids artificial covariance learning from absent measurements. When GNSS is reacquired, the R update and BMA-Q update resume from the last valid covariance state.

The proposed structure has three limiting cases. If M=1, D-VBAKF reduces to a conditional measurement-adaptive Kalman filter with fixed process covariance. If $\lambda_{R}=1$ and the BMA weights are fixed, it reduces to a fixed-covariance Kalman filter. If R is fixed and only the Q weights are updated, it reduces to a lightweight BMA-Q adaptive filter.

### 3.5. Implementation Details

#### 3.5.1. Initialization of State, Covariance and Hyperparameters

The initial error state is set according to the assumed MEMS INS/GNSS alignment and initialization uncertainty. The compared filters use the same initial covariance $P_{0|0}$. The initial measurement covariance ${\hat{R}}_{0}$ is set to the nominal GNSS covariance, and the initial BMA weights are uniform:

(33) $$w_{0}^{\left(m\right)}=\frac{1}{M},\;m=1,\ldots ,M.$$

The reported Monte Carlo comparisons use common numerical settings and paired trajectory and noise realizations across filters. The public-data comparisons use the settings in Table 1 without variant-specific tuning apart from the stated component switches. The turn-rate threshold 0.02 rad s^−1^ and motion-prior gains 5 and 0.5 are heuristic regularization coefficients rather than calibrated physical constants. The numerical public-data settings in Table 1 should not be read as a complete specification of the original simulation.

#### 3.5.2. Candidate *Q* Model Bank Design

The Q candidate model bank is designed as a scaled version of the nominal process covariance:

(34) $$\alpha_{m}\in \left\{0.1,0.3,1,3,10,30\right\}.$$

This grid covers both underestimated and overestimated process-noise conditions. Small candidates represent mild vehicle motion and stable MEMS behavior, whereas large candidates represent vibration, severe maneuvering, and increased sensor uncertainty. The grid is intentionally compact because the goal is online implementation rather than exhaustive offline covariance identification.

#### 3.5.3. R Bounds, Forgetting Factor and Numerical Stabilization

The mathrmR update uses a diagonal covariance structure with lower and upper bounds for each GNSS channel. The lower bound prevents the estimated measurement variance from falling below its nominal value; it does not ensure calibrated state uncertainty. The upper bound caps the estimate after an abnormal innovation.

The forgetting factor $\lambda_{R}$ balances smoothness and responsiveness. A value close to one gives stable R estimates but slower response to abrupt GNSS degradation. A smaller value improves tracking speed but increases covariance fluctuation. The reported comparisons use a common value within each protocol; the retained simulation records do not establish a separate tuning history.

#### 3.5.4. Entropy Floor and Weight Normalization

The BMA likelihood may concentrate most of the probability mass on a single Q candidate. Such concentration is useful when the dynamic condition is clear, but irreversible collapse makes recovery difficult if the noise condition later changes. Therefore, an entropy floor is applied after the likelihood-prior update. In the implementation, each weight is constrained to be no smaller than a small floor value, followed by renormalization:

(35) $$w_{k}^{\left(m\right)}=\frac{\max(w_{k}^{\left(m\right)},w_{\min})}{\sum_{j=1}^{M}\max\left(w_{k}^{\left(j\right)},w_{\min}\right)}.$$

This operation keeps every Q candidate available while still allowing the dominant model to receive most of the probability mass when supported by the data. Table 1 gives the complete numerical settings used for the added public-data experiment. The same initialization and numerical settings are used for all filter variants except the component switches that define each ablation.

**Table 1:** Implementation settings for the public-data stress test.

Item	Value or Rule
Error state and measurement	15 states (position, velocity, attitude, gyro bias, accelerometer bias); three-dimensional GNSS position update
Raw and processing rates	Raw IMU 200 Hz; consecutive increments summed to 20 Hz; GNSS and reference positions 1 Hz
Initial standard deviations	Position 1 m; velocity 0.2 m s^−1^; attitude 1°; gyro bias 50° h^−1^; accelerometer bias 500 mGal
Gauss–Markov correlation time	3600 s for gyro and accelerometer biases
GNSS antenna lever arm	(−0.073,0.302,0.087) m in the IMU forward-right-down frame
Q model bank	$\alpha_{m}\in \left\{0.1,0.3,1,3,10,30\right\}$; uniform initial weights
Conditional R update	$\lambda_{R}=0.90$; initial/lower standard deviations (0.6,0.6,1.2) m; upper standard deviations (5,5,10) m
BMA update	Weight-memory exponent β=0.92; prior width $\sigma_{q}=1.25$; numerical offset $\epsilon =10^{-12}$
Motion-informed prior	$\alpha_{\mathrm{prior}}=\mathrm{clip}[1+5\max(0,∥\Delta \theta /\Delta t∥-0.02)+0.5|∥\Delta v/\Delta t∥-g|,0.1,30]$
Weight constraint	$w_{\min}=0.01$ followed by normalization
Covariance stabilization	Joseph-form update; symmetry enforced after prediction and update; rotation matrix re-orthogonalized by singular-value decomposition
Compared variants	EKF-Fixed; CM-R only; BMA-Q only; D-VBAKF without motion prior; D-VBAKF without weight floor; full D-VBAKF
Software	Python 3.13, NumPy 2.4, pandas 3.0, and Matplotlib 3.10; single CPU thread

#### 3.5.5. Pseudocode of One Filtering Epoch

The procedure of one D-VBAKF epoch is summarized in Algorithm 1.
**Algorithm 1** One filtering epoch of the proposed D-VBAKF. 1:**Input** ${\hat{x}}_{k-1|k-1}$, $P_{k-1|k-1}$, ${\hat{R}}_{k-1}$, $w_{k-1}^{\left(m\right)}$ and $Q_{k}^{\left(m\right)}$. 2:Compute ${\bar{Q}}_{k-1}=\sum_{m}w_{k-1}^{\left(m\right)}Q_{k-1}^{\left(m\right)}$. 3:Perform the INS time update to obtain ${\hat{x}}_{k|k-1}$ and $P_{k|k-1}$. 4:If GNSS is unavailable, set ${\hat{x}}_{k|k}={\hat{x}}_{k|k-1}$, $P_{k|k}=P_{k|k-1}$, ${\hat{R}}_{k}={\hat{R}}_{k-1}$, and $w_{k}^{\left(m\right)}=w_{k-1}^{\left(m\right)}$, and then go to the next epoch. 5:If GNSS is available, compute $\nu_{k}=z_{k}-H_{k}{\hat{x}}_{k|k-1}$. 6:Update ${\hat{R}}_{k}$ using the conditional innovation moment and bounded fading-memory rule. 7:For each Q candidate, compute $P_{k|k-1}^{\left(m\right)}$, $S_{k}^{\left(m\right)}$ and $L_{k}^{\left(m\right)}$. 8:Update BMA weights using the log-domain likelihood-prior rule, apply the entropy floor, and normalize. 9:Compute ${\bar{Q}}_{k}=\sum_{m}w_{k}^{\left(m\right)}Q_{k}^{\left(m\right)}$.10:Perform the final measurement update and output ${\hat{x}}_{k|k}$, $P_{k|k}$, ${\hat{R}}_{k}$, ${\bar{Q}}_{k}$ and $w_{k}^{\left(m\right)}$.

For state dimension *n*, measurement dimension *d*, and Q-candidate number *M*, a dense implementation of the additional candidate covariance and likelihood operations beyond the shared Kalman update has the approximate cost

(36) $$O(Mdn^{2}+Md^{3}).$$

Because the Q-candidate likelihoods are independent, this step can be parallelized. The computational cost is evaluated directly using per-update runtime statistics in Section 4.

### 3.6. Public Vehicle Dataset and Controlled Stress-Test Protocol

The public-data stress test uses the consumer-grade ICM20602 sequence from the open Awesome GINS Dataset released by the Integrated and Intelligent Navigation Group at Wuhan University [36,37]. The 1617 s vehicle trajectory was collected in an open-sky industrial area and contains synchronized 200 Hz ICM20602 increments, 1 Hz RTK GNSS positions, and a post-processed navigation reference. The recorded mean and maximum speeds are 8.26 and 13.56 m s^−1^; the 95th-percentile and maximum absolute yaw rates are 11.77 and 28.37° s^−1^, respectively. The dataset-provided angle random walk, velocity random walk, bias standard deviations, body-frame lever arm, and one-hour correlation time are used without retuning. The noise parameters are reproduced in Table 2; the correlation time and lever arm are stated in Table 1.

Two controlled GNSS stressors are superimposed identically for all compared methods. First, twelve non-overlapping outages begin at 120 s and repeat every 120 s; durations cycle through 10, 30, and 60 s four times. Second, four 40 s intervals beginning at 180, 540, 900, and 1260 s add the deterministic north/east/down errors (2+4sin0.19u,−1+4cos0.13u,7sin0.11u) m, where *u* is time in seconds from interval onset. This explicit deterministic perturbation is a reproducible multipath-like stress test, not a claim that the original RTK data contain those errors. All remaining IMU, GNSS, and reference samples are the measured public data.

The public-data stress test and ablation use the same ICM20602 sequence. Reduced variants isolate conditional R adaptation, BMA-Q adaptation, the motion-informed prior, and the weight floor. The main comparison reports full-route 3D RMSE, 95th-percentile error, outage-window RMSE, NIS coverage, and update time; ablations report outage-window RMSE and endpoint error. The public-data update uses a three-dimensional position measurement, for which the appropriate nominal NIS reference is the central 95% chi-square interval with three degrees of freedom. Only aggregate coverage is retained, so the original per-epoch thresholding cannot be independently checked; the reported coverage is interpreted descriptively and cannot by itself verify covariance calibration. Repeated windows belong to one trajectory and are summarized descriptively, without an inferential real-data *p* value.

## 4. Experimental Validation and Results

### 4.1. Validation Design and Evaluation Metrics

The validation tests D-VBAKF under prescribed time-varying process and measurement noise in a 120 s simulation. The benchmark uses the 15-state error-state model of Section 3.2 and a six-dimensional GNSS position/velocity measurement at 10 Hz. The simulated Q(t) follows a prescribed dynamic envelope, while R(t) includes slow variation and a terminal degradation interval from 80 to 90 s when GNSS is available. This finite horizon evaluates transient behavior under controlled mismatch; it does not establish long-run steady-state performance.

Four filters are compared under identical trajectories and noise realizations. EKF-Fixed uses fixed nominal covariance matrices and represents conventional laboratory tuning. AMKF-Innov uses innovation-based process-covariance adaptation with fixed R. Joint-VB VBAKF performs coupled variational Bayesian Q/R adaptation. D-VBAKF is the evaluated decoupled method, combining a conditional covariance-matching R update with prior-regularized BMA-Q adaptation.

Retained simulation settings and results. The Monte Carlo validation uses 50 paired trials and GNSS outage durations of 10, 30, 60, and 90 s. The reported design specifies a 15-state model, 120 s horizon, six-dimensional GNSS position/velocity updates at 10 Hz, a terminal R degradation from 80 to 90 s when GNSS is available, and common trajectory, initial covariance, noise realization, and GNSS availability within each trial’s filter comparisons. The retained summaries include outage 3D position RMSE, endpoint and component errors, paired RMSE reduction and 95% intervals, win rate, no-outage Q-KL divergence and R error, R degradation delay, NIS coverage, and update time. A representative 60 s outage includes position, velocity, and attitude-error diagnostics. For baseline *b* and trial *i*, the paired reduction is $d_{i}={\mathrm{RMSE}}_{b,i}-{\mathrm{RMSE}}_{D\text{-}\mathrm{VBAKF},i}$. These exploratory intervals and tests are unadjusted for multiple comparisons. Table 3 instead gives intervals for each method’s mean RMSE, so paired comparisons are interpreted from Table 4. Table 4 reports $\bar{d}$ and the nominal normal-approximation interval $\bar{d}\pm 1.96s_{d}/\sqrt{50}$; the reported two-sided *p* values use the corresponding paired normal statistic.

Simulation information not recoverable from the retained materials. The original random-seed ledger, executable trajectory and exact Q(t)/R(t) schedules, trial-level records, complete innovation sequences, full posterior-covariance histories, and numerical simulator matrices and initial covariance values are unavailable. Table 1 reports numerical settings for the separate public-data stress test and does not fill these simulation gaps. Exact numerical reproduction of the original simulation is not possible from the retained materials. A newly specified and logged run would be needed to compute component-wise NEES and formal innovation-whiteness statistics. NIS is a measurement-space diagnostic and does not establish state-covariance consistency or unique Q/R allocation.

### 4.2. Navigation Accuracy Under Time-Varying Q/R and GNSS Outages

**Figure 2:**
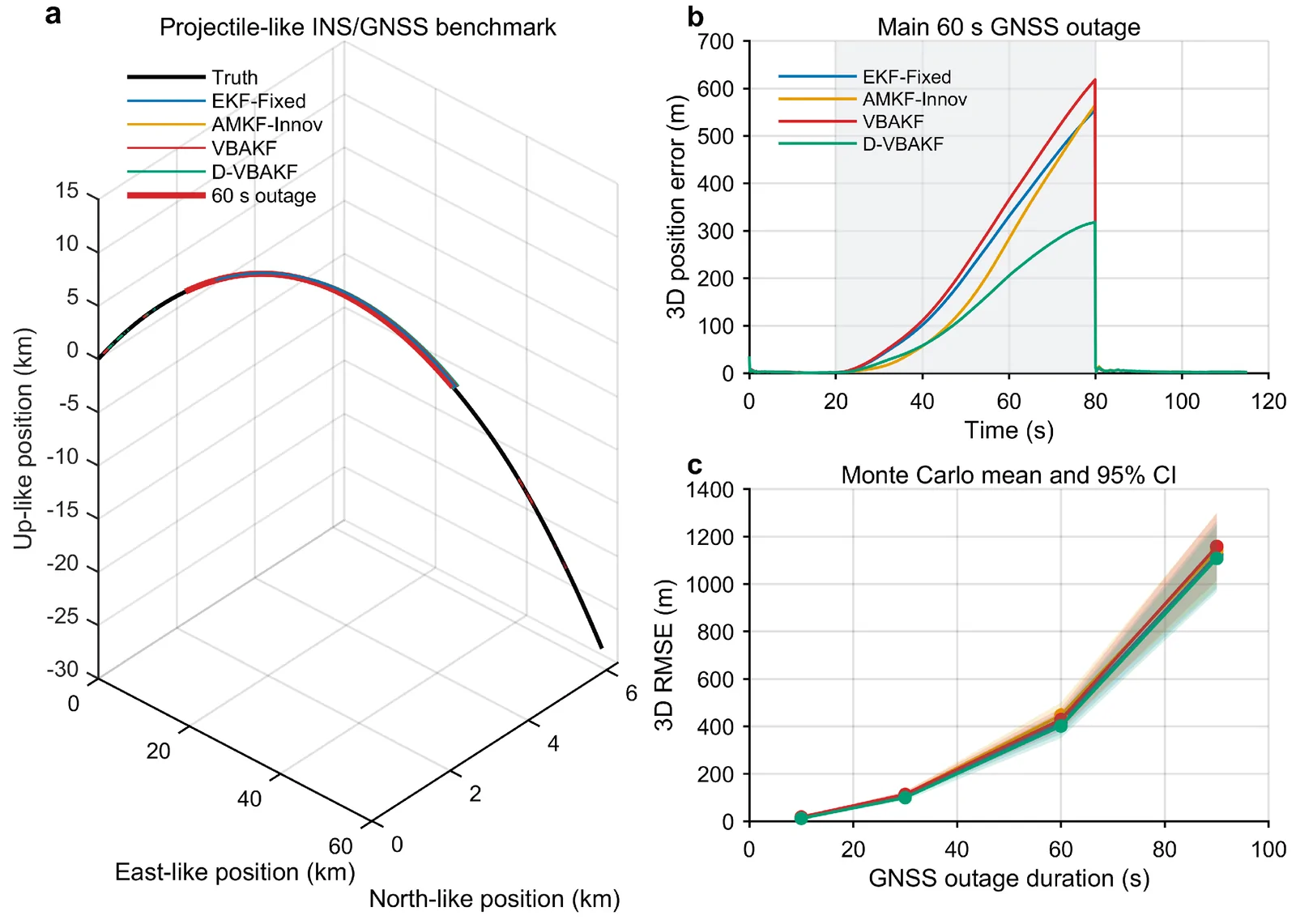
Navigation performance in the MEMS INS/GNSS benchmark. (**a**) Three-dimensional reference trajectory and the main 60 s GNSS outage interval. (**b**) Trial trajectory error in the main outage case. (**c**) Monte Carlo mean 3D RMSE and 95% confidence interval over outage duration.

Table 3 and Figure 2 report Monte Carlo outage 3D position RMSE and endpoint error. At 10 and 30 s, the mean reductions versus EKF-Fixed are 2.94 and 7.76 m, with paired 95% intervals of [1.59,4.29] and [1.51,14.02] m, respectively. The corresponding paired intervals versus the other two baselines are also positive. At 60 s, D-VBAKF has a mean RMSE of 401.52 m versus 414.81 m for EKF-Fixed, but the 13.29 m difference has a paired interval of [−11.32,37.91] m. At 90 s, all three paired intervals include zero. The longer-outage mean differences are therefore inconclusive for the indicated comparisons.

The main-text results show that D-VBAKF reduces most position components for 10 s and 30 s outages, whereas the gains become smaller and less uniform for 60 s and 90 s outages because inertial drift dominates during long GNSS interruptions.

### 4.3. Paired Statistical Significance Analysis

**Figure 3:**
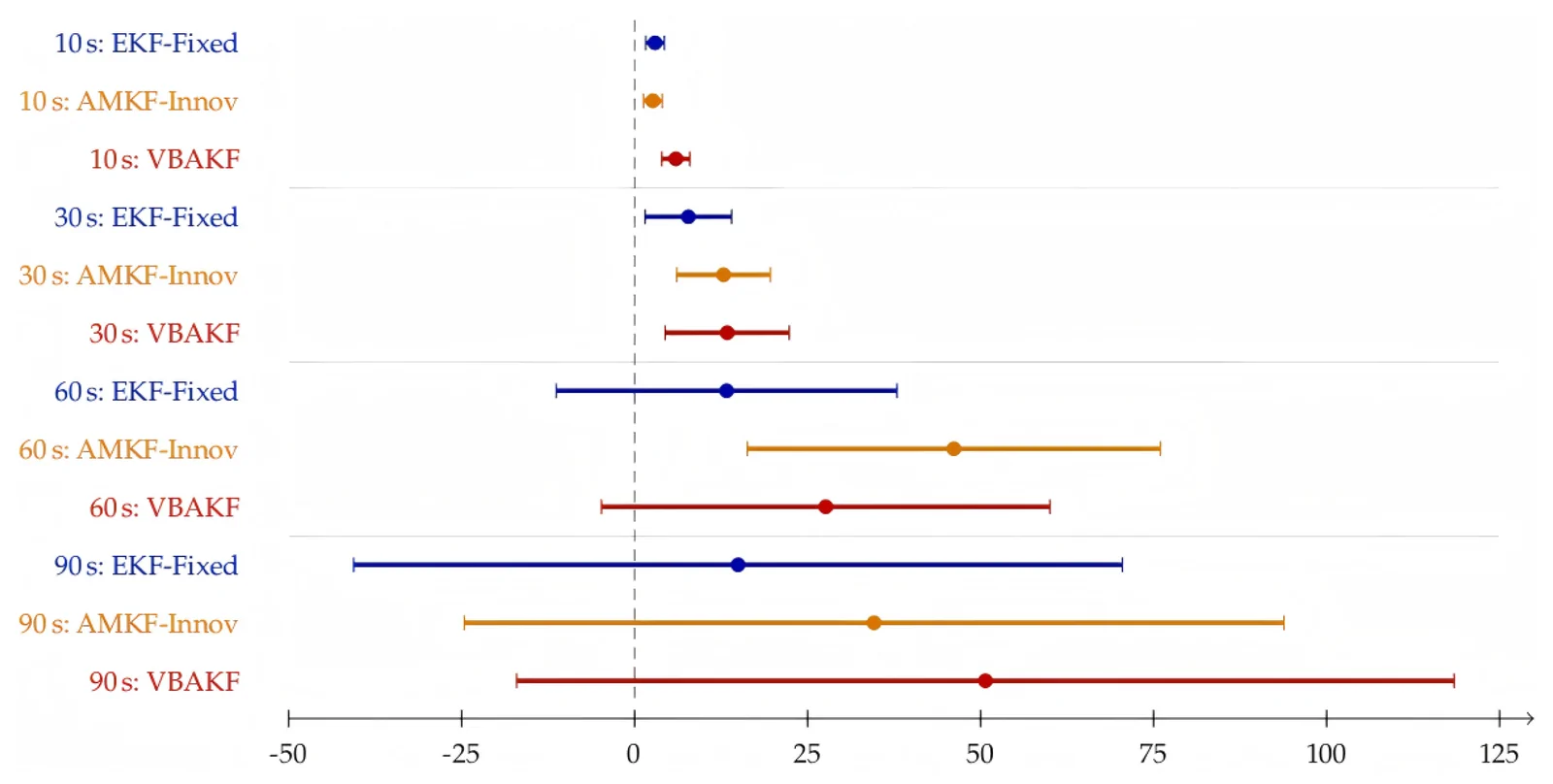
Paired mean 3D RMSE reducton (m).

Table 4 and Figure 3 show the paired RMSE reductions and their uncertainty. All reported 10 and 30 s intervals exclude zero on the positive side. At 60 s, the intervals versus EKF-Fixed and joint-VB VBAKF include zero, while the interval versus AMKF-Innov is positive. At 90 s, all three intervals include zero. Figure 3 displays the intervals against the zero-reduction reference; the individual-method means and standard deviations remain in Table 3.

Paired reductions in simulated outage 3D RMSE for D-VBAKF relative to each baseline. Dots show means, and horizontal bars show the nominal paired 95% intervals in Table 4; the dashed line indicates zero reduction. Positive values favor D-VBAKF. The intervals are unadjusted for multiple comparisons.

### 4.4. Known-Covariance Tracking in the Prescribed Simulation

Table 5 reports the no-outage simulation, in which prescribed Q(t) and R(t) vary while GNSS remains available. The full D-VBAKF implementation obtains Q-KL = 0.011 and R relative error = 0.173, compared with 1.227 and 0.297 for joint-VB VBAKF. These values describe tracking of the known schedules in this simulation. They do not isolate the incremental effect of BMA-Q or establish general Q/R identifiability; the true covariances are unavailable for the public-data test.

**Figure 4:**
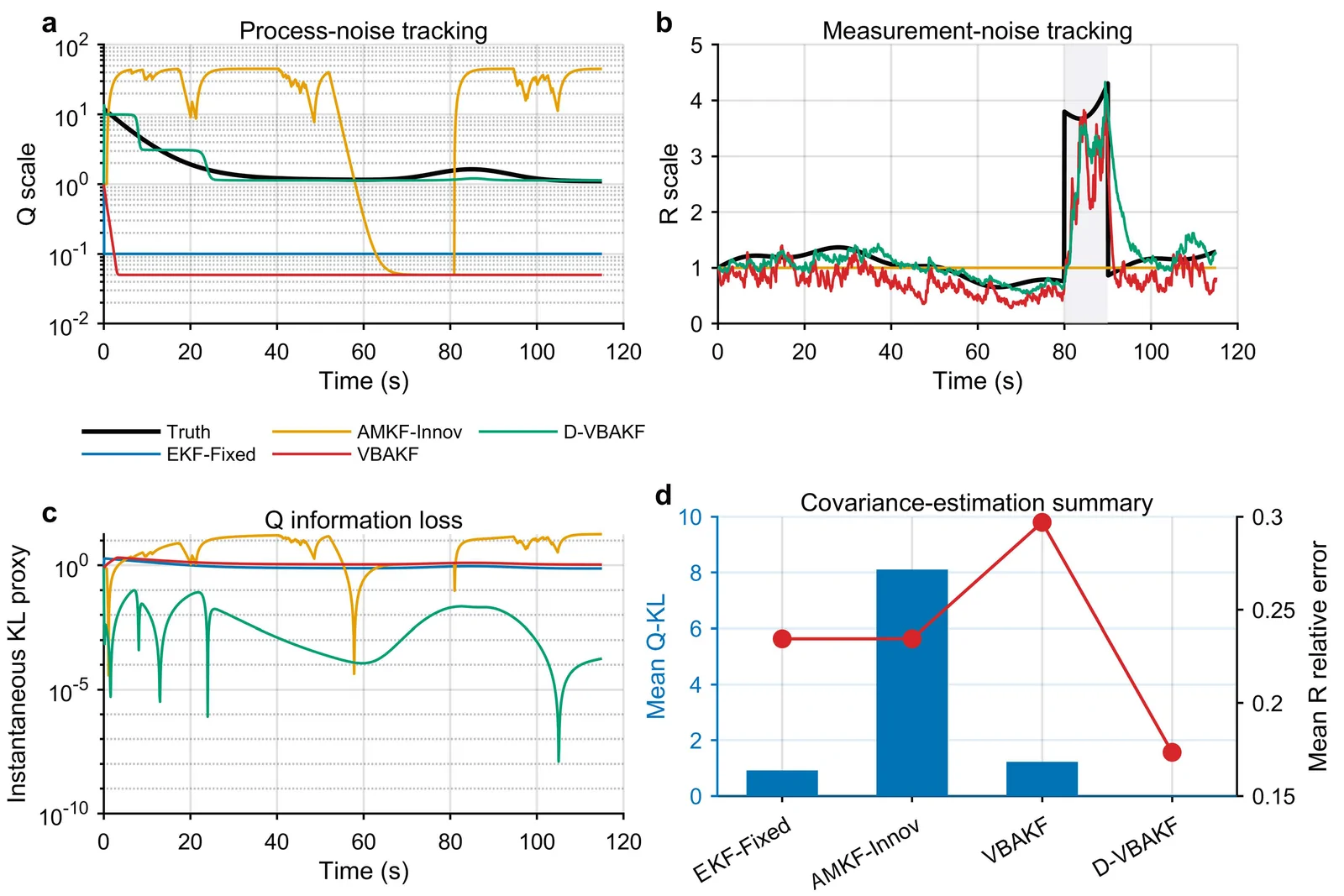
Process and measurement-covariance adaptation in the prescribed simulation. (**a**) Process-noise scale tracking. (**b**) Measurement-noise scale tracking with terminal degradation. (**c**) Instantaneous Q information-loss proxy. (**d**) Aggregate Q-KL and R relative-error summary.

As shown in Figure 4, the R degradation delay of D-VBAKF is slightly larger than that of joint-VB VBAKF in the no-outage tracking case. This indicates a trade-off between smoothing the estimated covariance and detecting an abrupt prescribed change. The reported delay does not characterize performance as a dedicated GNSS-degradation alarm. A change-point detector could be evaluated when rapid detection is required.

### 4.5. Measurement-Space NIS Analysis

Measurement-space innovation consistency is assessed using the normalized innovation squared (NIS),

(37) $$\epsilon_{k}=\nu_{k}^{T}S_{k}^{-1}\nu_{k}.$$

**Figure 5:**
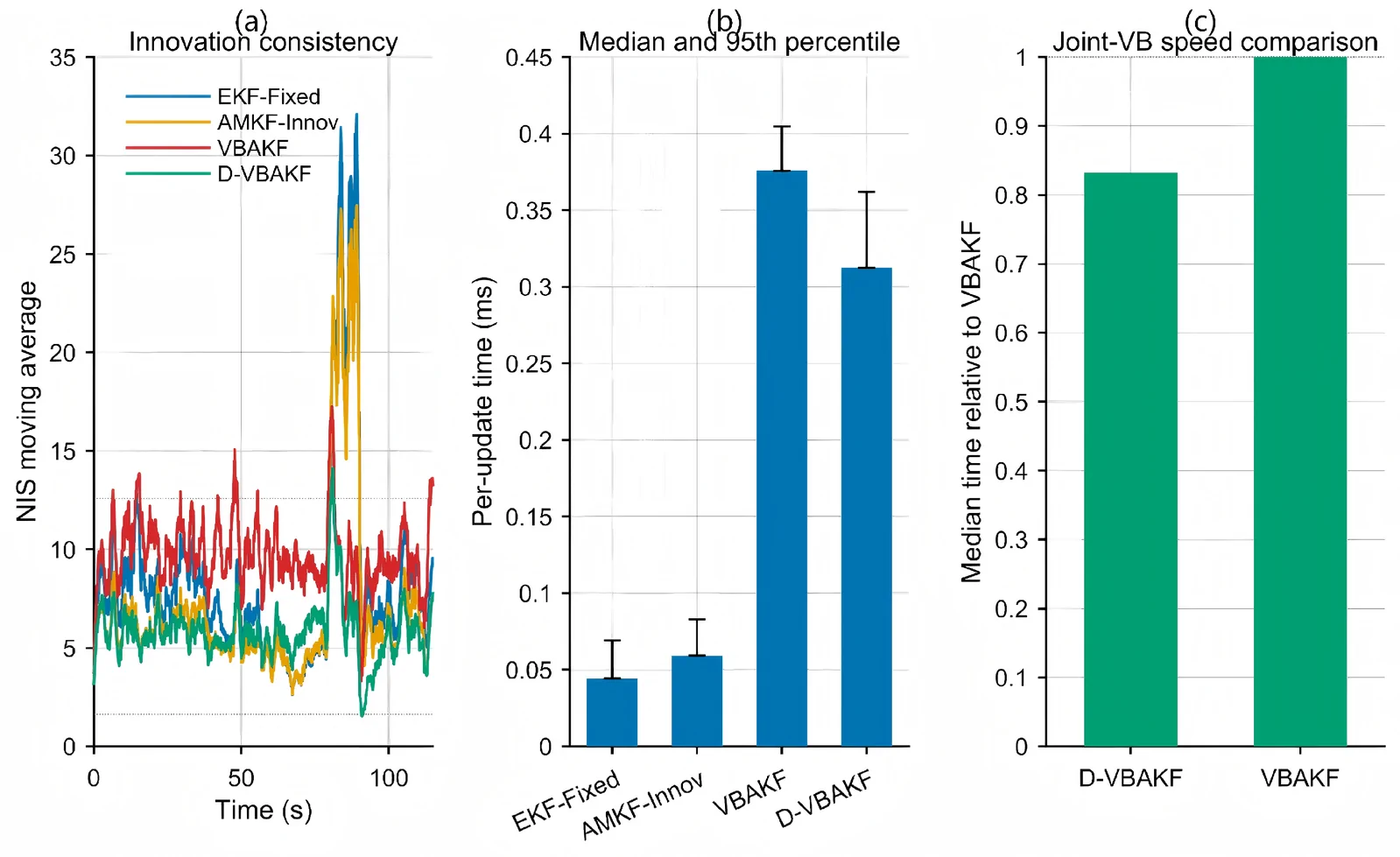
Innovation NIS and computational efficiency in the prescribed simulation. (**a**) Moving-average NIS; dotted lines show instantaneous-NIS chi-square reference values for context, not acceptance limits for the moving average. (**b**) Median and 95th-percentile update time. (**c**) Median update time relative to joint-VB VBAKF.

For the six-dimensional simulated GNSS measurement, the nominal reference is the central 95% chi-square interval with six degrees of freedom. Table 6 reports the fraction of NIS values inside those bounds. D-VBAKF has the highest reported fraction, 0.898, versus 0.780, 0.836, and 0.720 for EKF-Fixed, AMKF-Innov, and VBAKF; 0.898 remains below the nominal 0.95. This is a measurement-space diagnostic only: an adapted R can compensate for an incorrect predicted-state covariance, so NIS alone cannot verify Q/R allocation or state-covariance consistency. The public-data NIS coverage is considered separately because its GNSS update has three position components and the data follow one route. The verification results are shown in Figure 5.

NEES would test state-estimation error against the posterior covariance, while formal innovation-whiteness tests require complete innovation sequences. Full $P_{k|k}$ and innovation histories were not retained for all Monte Carlo trials, so neither component-wise/full-state NEES nor formal whiteness statistics are reported. The simulated Q-KL and R-error metrics, rather than NIS alone, support the covariance-tracking comparison.

### 4.6. Interpretability of BMA-Based Q Adaptation

The BMA weights describe which prescribed process-noise scales are favored by the update. In the representative no-outage simulation trajectory, the dominant model shifts from a large-Q candidate during the early high-dynamic phase to moderate and nominal candidates as the trajectory becomes milder. These weights document model-bank behavior in this simulation; they do not identify a physical process covariance or demonstrate a separate navigation benefit from BMA-Q.

### 4.7. Ablation, Robustness and Sensitivity

The simulated baselines compare different adaptation structures: EKF-Fixed uses nominal covariances, AMKF-Innov adapts process uncertainty with fixed R, and joint-VB VBAKF uses coupled covariance learning. Their Q-KL and R-error values provide a comparison on the prescribed schedules. NIS is retained as a separate measurement-space diagnostic. The public-data component ablation below tests the contributions of the implemented design elements on one route.

Across 10, 30, 60, and 90 s outages, D-VBAKF has the lowest reported mean 3D RMSE, while all three paired intervals are positive only for the 10 and 30 s cases. The 60 s comparisons with EKF-Fixed and joint-VB VBAKF and all 90 s comparisons include zero. Covariance adaptation cannot remove unaided inertial drift during long GNSS losses. The sensitivity of the scalar Q grid, R forgetting factor, motion-prior coefficients, and weight floor requires validation beyond the reported scenarios.

### 4.8. Computational Efficiency

Table 7 reports computational time in the evaluated implementation. The median D-VBAKF update takes 0.313 ms. It is slower than EKF-Fixed and AMKF-Innov because of the conditional R moment update and BMA likelihood calculation, and about 16.8% faster than joint-VB VBAKF. This supports online feasibility for the reported configuration.

The reported update times reflect two implementation choices. First, D-VBAKF uses a shared state estimate rather than a full bank of parallel filters. Second, the Q-candidate likelihoods are independent and can be parallelized. The measured times support online feasibility for the reported implementation and workload.

### 4.9. Public-Data Stress Test on a Consumer-Grade MEMS IMU

**Figure 6:**
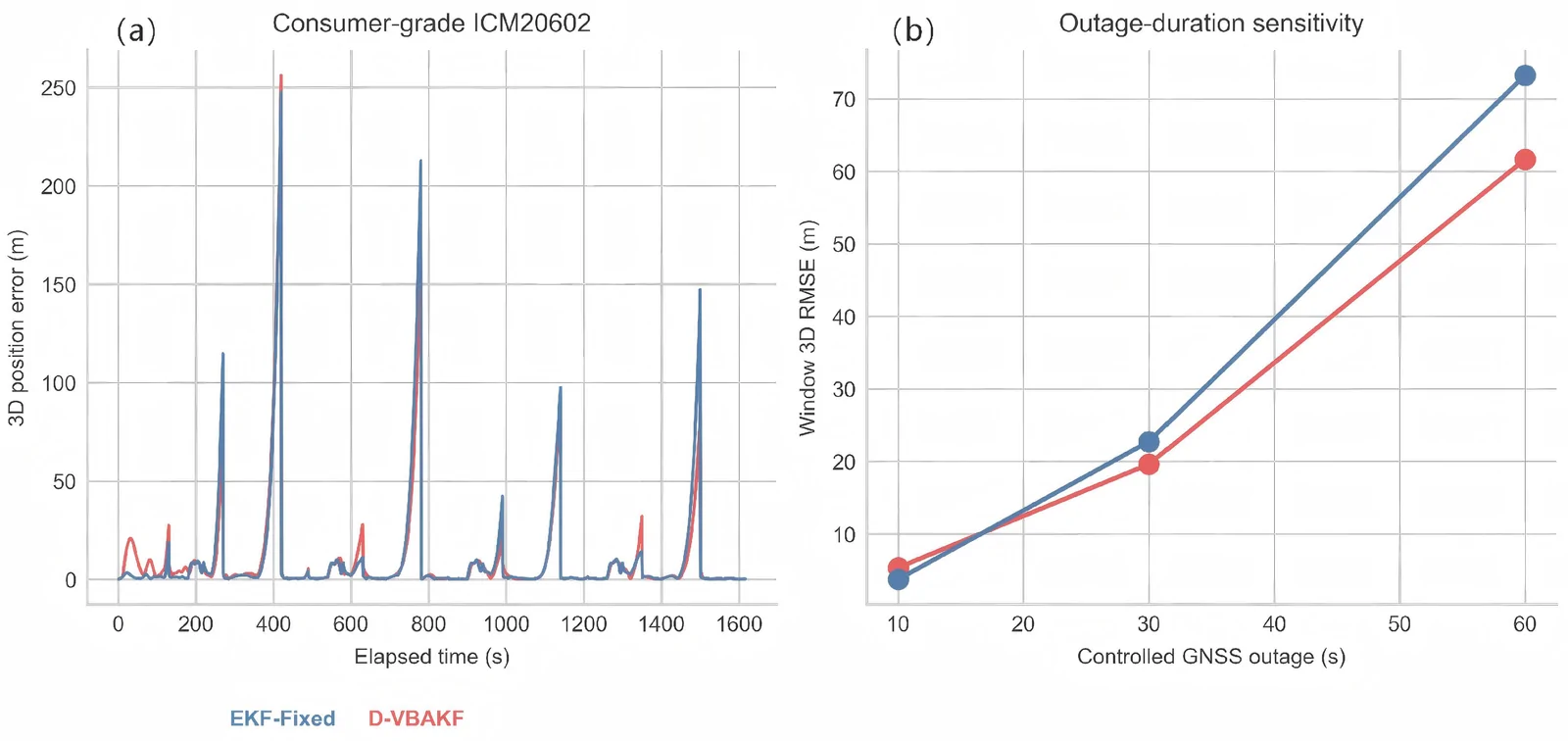
Single-trajectory ICM20602 stress test. (**a**) Three-dimensional position error over the 1617 s drive. (**b**) Descriptive mean RMSE for four controlled outage windows at each duration on that same drive. Windows are repeated segments, not independent trajectories; no between-drive confidence interval is available. Multipath-like GNSS perturbations are deterministic and identical for both methods.

Table 8 and Figure 6 report a controlled stress test on one mainly open-sky ICM20602 trajectory. With the same deterministic GNSS perturbations, full-route 3D RMSE is 30.77 m for EKF-Fixed and 27.19 m for D-VBAKF, an observed 11.6% within-drive difference. The 95th-percentile error is 67.00 versus 50.96 m, and the descriptive mean outage-window RMSE is 33.23 versus 28.86 m. NIS coverage changes by 0.010, from 0.626 to 0.636; both values are below the nominal 0.95 coverage target and do not establish covariance calibration. Median update time is 0.054 versus 0.195 ms. These comparisons describe the imposed-stressor configuration on this one route; the repeated windows do not provide independent-drive uncertainty estimates.

### 4.10. Component Ablation on the Consumer-Grade IMU

Across twelve controlled GNSS outage windows on one ICM20602 drive, the CM-R-only variant has a mean 3D RMSE of 28.19 m, compared with 28.86 m for the full D-VBAKF method. BMA-Q alone has a mean of 33.98 m, compared with 33.23 m for EKF-Fixed. Removing the motion-informed prior yields 38.89 m; removing the weight floor yields 28.69 m. Table 9 also reports endpoint errors and window standard deviations. The windows are repeated segments of one drive.

## 5. Discussion

In the prescribed no-outage simulation, the full method tracks the known Q(t) and R(t) schedules with Q-KL = 0.011 and R relative error = 0.173. The simulation’s paired navigation intervals relative to all three baselines are positive at 10 and 30 s. At 60 s, the intervals versus EKF-Fixed and joint-VB VBAKF include zero; all 90 s intervals include zero. Figure 3 makes this uncertainty visible. The public-data comparison uses one mainly open-sky vehicle trajectory with deterministic GNSS degradations and repeated outage windows. Its 30.77-to-27.19 m full-route RMSE difference is a descriptive observation on that route, not a population-level accuracy estimate.

Navigation RMSE and covariance calibration address different questions. In the public-data test, NIS coverage differs by 0.010 between D-VBAKF and EKF-Fixed (0.636 versus 0.626), with both values below the nominal 0.95. The result does not demonstrate calibrated measurement or state covariance. An adapted R can compensate for a misspecified predicted-state covariance, so NIS alone cannot establish correct Q/R allocation. Q-KL = 0.011 is measured only against the known Q(t) of the prescribed simulation; no true Q or R is available for an analogous real-data tracking claim.

Innovation-based filters use innovation statistics to tune the stochastic model and face a window–length trade-off. Joint-VB methods impose probabilistic structure but may require additional temporal or structural information to distinguish freely varying Q and R at one epoch. Multiple-model methods represent process uncertainty through candidate models. D-VBAKF combines a conditional R moment approximation with a compact scaled Q bank and a shared state estimate, keeping the measured update time moderate.

**Figure 7:**
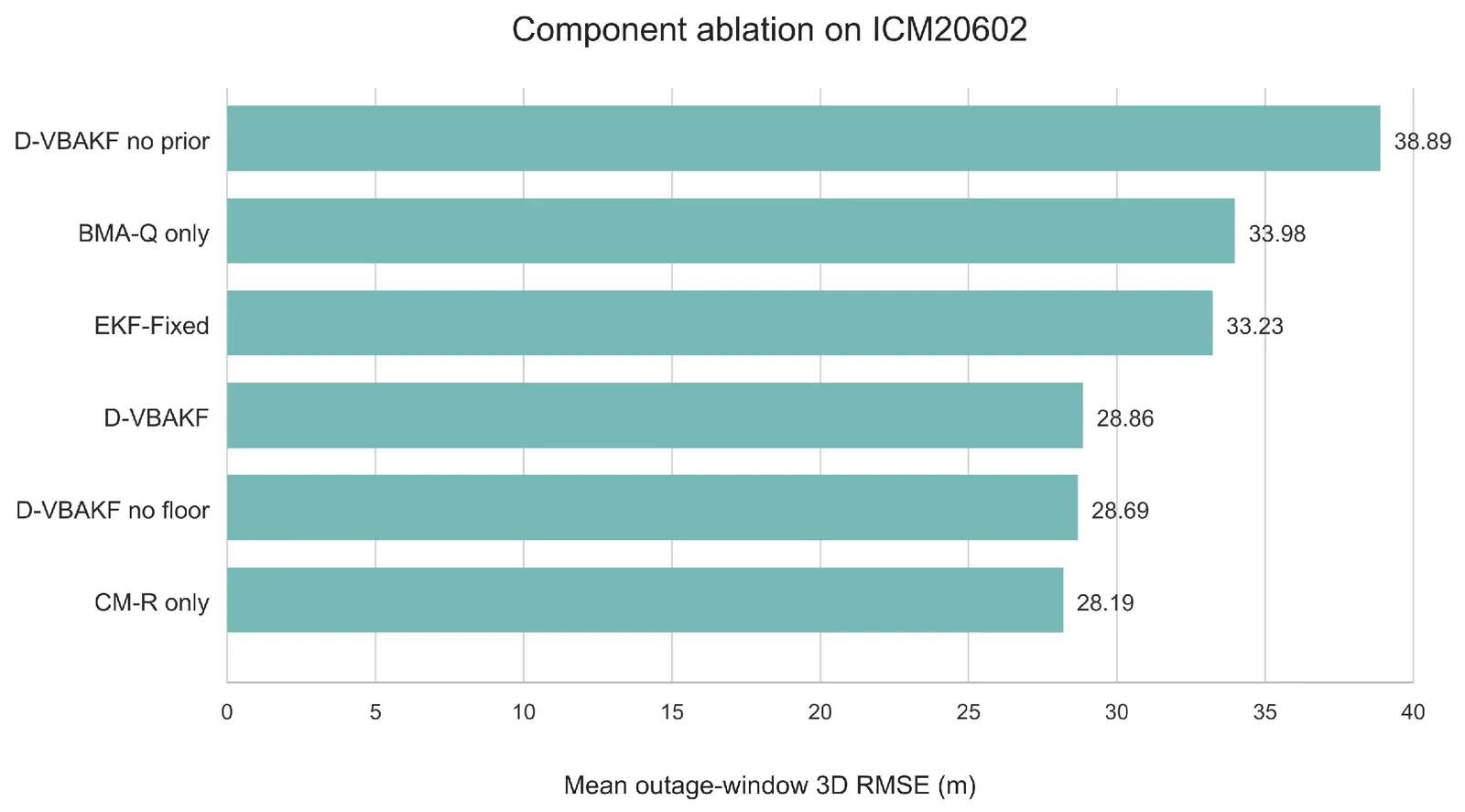
Component ablation across twelve controlled GNSS outage windows on one ICM20602 drive. Bars show the mean 3D RMSE; the corresponding means and standard deviations are reported in Table 9. All windows are repeated segments of the same drive.

The BMA weights are preferences among prescribed scales $\alpha_{m}$ of $Q_{0}$. The resulting covariance ${\bar{Q}}_{k}=\left(\sum_{m}w_{k}^{\left(m\right)}\alpha_{m}\right)Q_{0}$ has only one effective scale degree of freedom; it cannot represent a change in covariance shape. Its algorithmic role is to bound process-noise scaling and expose model weights; the ablation shows no measured accuracy gain from adding BMA-Q to R adaptation on this drive. Table 9 and Figure 7 show that the R-only variant has lower mean window RMSE than the full method, whereas BMA-Q alone has a 0.75 m higher mean RMSE than EKF-Fixed. Removing the motion-informed prior increases the mean by 10.03 m relative to the full method, indicating dependence on its empirical coefficients in this test. The retained materials do not document how those coefficients were originally selected, and no parameter-wise sensitivity analysis is reported. The twelve correlated windows do not support decomposing the observed difference into independent component effects.

This study has several limits. The public data come from one mainly open-sky ICM20602 route; imposed stressors are not naturally labeled urban-canyon events, and repeated windows are not independent drives. The public-data NIS indicates measurement-space undercoverage, but without true Q/R and complete posterior-covariance histories, the source of miscalibration and state-covariance calibration cannot be determined. The R update cannot run during a complete GNSS outage because no innovation exists, and the diagonal R model omits realistic cross-channel correlations. The Q bank adapts only overall scale, with sensitivity to heuristic motion-prior coefficients. The 120 s simulation does not establish steady-state behavior. The original seed ledger, complete executable trajectory and Q(t)/R(t) schedules, full posterior-covariance histories, and complete innovation histories are unavailable. Exact reproduction of the original simulation is not possible from the retained materials; NEES and formal whiteness testing would require complete logging in a new experiment.

Future work should test more flexible Q covariance shapes, assess motion-prior sensitivity, and combine adaptation with abnormal-measurement detection. Independent drives with naturally degraded GNSS, longer simulations, and complete state-covariance and innovation logging are needed for broader validation.

## 6. Conclusions

The evaluated filter and experiments yield the following findings Method D-VBAKF combines bounded fading-memory covariance matching for R with motion-regularized BMA over six scalar multiples of $Q_{0}$. The resulting process covariance changes scale but not shape.Prescribed simulation. In 50 paired trials, 3D position RMSE reductions relative to EKF-Fixed were 2.94 m (nominal paired 95% interval [1.59,4.29] m) at 10 s and 7.76 m ([1.51,14.02] m) at 30 s. The corresponding intervals versus the other two baselines were also positive. At 60 s, intervals versus EKF-Fixed and joint-VB VBAKF included zero; all three 90 s intervals included zero. In a separate no-outage simulation with prescribed covariances, Q-KL was 0.011 and R relative error was 0.173.

Single-drive stress test. On one public ICM20602 trajectory with imposed GNSS stressors, full-route 3D RMSE was 27.19 m for D-VBAKF and 30.77 m for EKF-Fixed. Across twelve repeated outage windows, mean RMSE was 28.19 m for conditional R adaptation alone, 28.86 m for the full method, 33.98 m for BMA-Q alone, and 33.23 m for EKF-Fixed. NIS coverage was 0.636 for D-VBAKF and 0.626 for EKF-Fixed, both below the nominal 0.95.

Future evaluation should include independent drives with naturally degraded GNSS, motion-prior sensitivity analysis, and complete posterior-covariance and innovation records.

## Figures and Tables

**Table 2:** ICM20602 characteristics used by the public-data filter.

IMU	Grade	ARW (°/$\sqrt{h}$)	VRW (m s^−1^/ $\sqrt{h}$)	Gyro Bias SD (°/h)	Accel. Bias SD (mGal)
ICM20602	Consumer chip	0.2	0.2	200	1000

**Table 3:** Outage 3D position RMSE and endpoint error with 95% confidence intervals.

Outage (s)	Method	Mean 3D RMSE (m)	SD (m)	95% CI Low (m)	95% CI High (m)	Mean Endpoint Error (m)
10	EKF-Fixed	15.08	5.84	13.46	16.70	32.23
10	AMKF-Innov	14.77	6.04	13.09	16.44	31.64
10	VBAKF	18.09	7.67	15.96	20.21	37.78
10	D-VBAKF	12.14	4.73	10.83	13.46	26.44
30	EKF-Fixed	107.82	43.50	95.76	119.87	244.24
30	AMKF-Innov	112.91	46.47	100.03	125.79	252.90
30	VBAKF	113.43	45.92	100.70	126.16	254.72
30	D-VBAKF	100.05	44.43	87.74	112.37	228.90
60	EKF-Fixed	414.81	171.55	367.26	462.36	939.19
60	AMKF-Innov	447.65	190.35	394.89	500.42	998.67
60	VBAKF	429.13	174.16	380.86	477.41	965.84
60	D-VBAKF	401.52	182.84	350.84	452.20	913.07
90	EKF-Fixed	1123.06	515.88	980.07	1266.06	2450.64
90	AMKF-Innov	1142.72	557.28	988.25	1297.19	2485.21
90	VBAKF	1158.80	514.60	1016.16	1301.44	2512.31
90	D-VBAKF	1108.11	508.92	967.04	1249.17	2415.03

**Table 4:** Paired RMSE reduction tests for D-VBAKF. Positive reduction means lower D-VBAKF RMSE. Intervals and two-sided normal-approximation tests are nominal and unadjusted for multiple comparisons.

Outage (s)	Comparison	Mean RMSE Reduction (m)	95% CI Low (m)	95% CI High (m)	Paired z-Test *p* Value	Win Rate
10	D-VBAKF vs. EKF-Fixed	2.937	1.589	4.285	1.96 × 10^−5^	68.0%
10	D-VBAKF vs. AMKF-Innov	2.621	1.261	3.981	1.58 × 10^−4^	70.0%
10	D-VBAKF vs. VBAKF	5.945	3.914	7.975	9.55 × 10^−9^	78.0%
30	D-VBAKF vs. EKF-Fixed	7.762	1.509	14.015	0.015	68.0%
30	D-VBAKF vs. AMKF-Innov	12.853	6.084	19.622	1.98 × 10^−4^	76.0%
30	D-VBAKF vs. VBAKF	13.376	4.402	22.350	0.00348	62.0%
60	D-VBAKF vs. EKF-Fixed	13.294	−11.321	37.909	0.290	58.0%
60	D-VBAKF vs. AMKF-Innov	46.137	16.285	75.989	0.00245	62.0%
60	D-VBAKF vs. VBAKF	27.618	−4.787	60.022	0.0948	62.0%
90	D-VBAKF vs. EKF-Fixed	14.955	−40.584	70.495	0.598	60.0%
90	D-VBAKF vs. AMKF-Innov	34.608	−24.629	93.846	0.252	54.0%
90	D-VBAKF vs. VBAKF	50.695	−17.042	118.431	0.142	62.0%

**Table 5:** Comparison with prescribed Q and R schedules in the no-outage simulation.

Method	Q-KL	R Relative Error	R Detection Delay (s)
EKF-Fixed	0.918	0.235	N/A
AMKF-Innov	8.107	0.235	N/A
VBAKF	1.227	0.297	3.763
D-VBAKF	0.011	0.173	3.890

**Table 6:** Fraction of simulated NIS values within the nominal central 95% interval.

Method	NIS Interval Coverage
EKF-Fixed	0.780
AMKF-Innov	0.836
VBAKF	0.720
D-VBAKF	0.898

**Table 7:** Per-update runtime statistics.

Method	Mean (ms)	Median (ms)	P95 (ms)	Maximum (ms)	Median/EKF	Median/ VBAKF
EKF-Fixed	0.049	0.044	0.069	0.120	1.000	0.118
AMKF-Innov	0.064	0.059	0.083	0.124	1.336	0.158
VBAKF	0.382	0.376	0.405	0.665	8.482	1.000
D-VBAKF	0.319	0.313	0.362	0.600	7.057	0.832

**Table 8:** Descriptive results for one ICM20602 trajectory under controlled GNSS degradation and outages. The nominal NIS coverage target is 0.95.

IMU	Method	3D RMSE (m)	P95 Error (m)	NIS Coverage	Median Update (ms)
ICM20602	EKF-Fixed	30.77	67.00	0.626	0.054
ICM20602	D-VBAKF	27.19	50.96	0.636	0.195

**Table 9:** ICM20602 component ablation across twelve controlled outage windows from one drive. Values are descriptive mean ± SD. Difference versus full is variant mean RMSE minus full-method mean RMSE; negative values indicate lower error.

Variant	Window 3D RMSE (m)	Difference vs. Full (m)	Mean Endpoint Error (m)	Windows
EKF-Fixed	33.23±35.52	+4.37	76.11	12
CM-R only	28.19±30.74	−0.67	64.83	12
BMA-Q only	33.98±33.50	+5.12	77.55	12
D-VBAKF without motion prior	38.89±43.86	+10.03	89.01	12
D-VBAKF without weight floor	28.69±30.88	−0.17	65.92	12
D-VBAKF	28.86±31.23	0.00	66.26	12

## Data Availability

The public-data test data are publicly available from the Wuhan University i2Nav Awesome GINS Dataset at https://github.com/i2Nav-WHU/awesome-gins-datasets (accessed on 30 September 2026). The analysis uses the ICM20602 inertial data, GNSS RTK positions, and corresponding reference trajectory. The deterministic GNSS perturbations and numerical settings are described in the controlled stress-test protocol and Table 1. A script for reproducing Figure 7 from the means in Table 9 accompanies the source files; original simulation and filtering code and derived CSV files are unavailable.
